# Identified plasma proteins related to vascular structure are associated with coarctation of the aorta in children

**DOI:** 10.1186/s13052-020-00830-7

**Published:** 2020-05-19

**Authors:** Siyu Ma, Junqiang Zheng, Yang Xu, Zhaocong Yang, Yu Zhu, Xiaoqi Su, Xuming Mo

**Affiliations:** Department of Cardiothoracic Surgery, Children’s Hospital of Nanjing Medical University, 72 Guangzhou Road, Nanjing, 210008 China

**Keywords:** Coarctation of the aorta, Plasma proteins, Vascular structure, FBLN1, ALS

## Abstract

**Background:**

Coarctation of the aorta (CoA), presenting with local stenosis of the aorta is involved in many cardiovascular processes. However, there has been little research on the mechanism of coarctation of the aorta.

**Methods:**

Altered proteins were identified by isobaric tag for relative and absolute quantitation (iTRAQ) technology in 8 participants, and further analysed by heatmap, Gene Ontology (GO), Kyoto Encyclopedia of Genes and Genomes pathway (KEGG) and Search Tool for the Retrieval of Interacting Gene (STRING). Of these, two vascular structure-related proteins were further validated by using enzyme-linked immunosorbent assay (ELISA) in a new cohort of CoA patients.

**Results:**

39 differentially expressed plasma proteins were first identified in patients with coarctation of the aorta by iTRAQ. Of these, fibulin-1 (FBLN1) and insulin-like growth factor-binding protein complex acid labile subunit (ALS) were considered candidates and further validation also showed that the level of FBLN1 in the CoA group (8.92 ± 2.36 μg/ml) was significantly higher compared with control group (6.13 ± 1.94 μg/ml), and the level of ALS in CoA children (348.08 ± 216.74 ng/ml) was significantly lower than the level in normal children (619.46 ± 274.08 ng/ml).

**Conclusions:**

The differentially expressed proteins identified in the plasma from CoA patients indicated that they may play critical roles in CoA and that they could potentially be utilized as biomarkers for diagnosis. Altered vascular related proteins were associated with COA. These results provide a foundation for further understanding and studying the aetiology and pathogenesis of coarctation of the aorta.

## Introduction

Coarctation of the aorta (CoA) is a diffuse arteriopathy, shown as a local stenosis of the aortic lumen on account of medial wall in-folding and thickening of aortic wall tissue [1]. Three out of 1000 live births had isolated CoA with a male to female ratio of 1.5:1 [2]. CoA is a common congenital heart disease (CHD), accounting for 5–8% of CHD patients [3, 4]. In addition, CoA is usually complicated with other developmental abnormalities of the heart and blood vessels, such as ventricular septal defect, aortic arch hypoplasia, patent ductus arteriosus and bicuspid aortic valve [1]. Patients with CoA usually clinically present with arterial systolic hypertension that is generally > 20 mmHg higher in the upper extremities than in the lower extremities, because of the different haemodynamics between the two sides of narrowing area [2]. In addition, the reason for the hemodynamic changes caused by aortic coarctation was that CoA was involved in multi-cardiovascular processes.

The influence of CoA in the cardiovascular system not only manifested as differences in the blood pressure of the upper and lower limbs, but was also nonnegligible, which resulted from haemodynamic changes caused by stenosis. Many studies have shown, that CoA not only causes abnormalities in anatomy, but also causes changes in pathology and physiology [3–6]. Stenosis is usually located distal to the start of the left subclavian artery and proximal to the ductus arteriosus or ligament [4]. High blood pressure at a narrow location proximal to the heart increased the left ventricle (LV) load, altering LV structure and function gradually [5]. In addition, CoA also influenced the aortic arch and led to wider vasculopathy [7], such as aortic rupture or dissection, adolescent hypertension, coronary and cerebral artery diseases [8, 9]. A study by autopsying 304 unoperated patients with CoA has shown, that 90% of patients with CoA, who had survived the first two years of life, died by 55 [10]. Therefore, it was of great significance to investigate the aetiology of CoA.

Vascular alteration was considered a vital event in the process of CoA. Physiological and pathological studies may provide a direction for us to study the aetiology of CoA. A study on changes in narrowed aorta has shown that cystic changes characteristically existed in aortic media with elastin fragmenting and collagen deposition increasing [6]. For improving the prognosis of CoA patients, however, most of the research has focused on how to relieve aortic narrowing via surgery [10–13]. Further molecular studies on the vasculature may contribute to the understanding of the aetiology of CoA. Some studies have focused on genetic or proteomic studies to uncover the pathogenesis of CoA. Of these, natriuretic peptide receptor C (NPR-C) was identified as being correlated with CoA by using RNA sequencing in aortic tissue from humans and by validating the results in human aortic endothelial cells [14]. In addition, by analysing aortic tissue, a proteomic/phosphoproteomic study on CoA patients with bicuspid and normal tricuspid aortic valve has shown that the change in proteins related to elastin, oxidative stress and inositol signalling pathways may influence the risk of cardiovascular events [15]. However, this study did not investigate the proteins that may increase the risk of having CoA. However, there is no research on human CoA-related plasma proteins by using the isobaric tag for relative and absolute quantitation (iTRAQ) method, which could more exactly reflect the levels of protein expression [16]. Plasma proteins related to CoA may uncover the possible genetic deficiency and mechanisms of CoA, which have strong clinical significance.

In our study, we identified two differentially expressed proteins (DEPs) related to vascular structure from the plasma of CoA children compared with those in the plasma of normal children by using the iTRAQ method. These differential proteins may be expected to contribute to promote the understanding of the pathogenesis and aetiology of CoA.

## Materials and methods

### Study population and sample preparation

From January 2012 to December 2017, we enrolled 54 CoA patients treated in the department of cardiothoracic surgery of Children’s Hospital of Nanjing Medical University. The Ethics Committee (Institutional Review Board) of the hospital approved this study protocol (201806182–1). The guardians of CoA children have understood and approved the informed consent. Those CoA children were all evaluated by routine clinical assessments, diagnosed by computed tomography angiography and/or preoperative echocardiography and treated by corrective surgery. Additionally, we also recruited 54 age- and gender-matched children from the Han population as controls in the study. The control group was selected from normal children after the physical examination or the CHD screening programme in the hospital. It was confirmed that all controls do not have cardiac diseases by clinical screening or additional echocardiography.

We collected CoA children’s blood before they performed surgery. Each blood sample mixed with ethylene diamine tetraacetic acid was harvested in collection tubes and centrifuged at the speed of 1500 g for 10 min, which ensured blood cells could be separated from the plasma. Then, we collected plasma and divided it into aliquots. Each was stored frozen at − 80 °C until we carrying out the analysis.

### Depletion of high-abundance proteins

The high abundant proteins in plasma samples collecting from CoA and normal children were processed to deplete by using the ProteoMiner™ Protein Enrichment Small-Capacity Kit (Bio-Rad, China).

### Enzymatic hydrolysis and desalination

An equal amount of protein from each sample was used for trypsin digestion. We digested 100 μg protein by using trypsin. First, after using tetraethylammonium bromide (TEAB) to dilute proteins five times, trypsin was added into diluted proteins with trypsin and a protein mass ratio of 1:50. The enzyme solution was hatched for enzymolysis overnight at 37 °C. After enzymolysis, peptides were desalinated with a C18 column and freeze-dried in vacuum.

### Isobaric tags for relative and absolute quantitation (iTRAQ) labelling

The peptides were dissolved with 0.5 m TEAB and labelled according to the iTRAQ-8 standard kit (SCIEX) instructions. The samples were labelled and mixed. Then, the mixed peptides were graded and separated using the Ultimate 3000 HPLC system (Thermo DINOEX, USA). The column was Durashell C18 (5 μm, 100 A, 4.6 × 250 mm). The acetonitrile (ACN) concentration gradually increased under alkaline conditions to achieve the separation of the peptide segment, and one tube was collected every minute with a flow rate of 1 ml/min. A total of 42 secondary fractions were collected and combined into 12 fractions, which were desalted and vacuum dried on the Strata-X column.

### Analysis by liquid chromatography-mass spectrometry

Mass spectrometry data were collected using the TripleTOF 5600 plus liquid mass combined system (SCIEX, USA). The sample was dissolved in 2% acetonitrile /0.1% methylic acid and analysed by the TripleTOF 5600 plus mass spectrometer coupled to the Eksigent nano LC system (SCIEX, USA). The polypeptide solution was added to the C18 capture column (5 μm, 100 μm × 20 mm) at a time gradient of 90 min with a flow rate of 300 nL/min to elute in a C18 analysis column (3 μm, 75 μm × 150 mm). Two mobile phases in gradient elution are buffer A (98 2% acetonitrile / 0.1% formic acid/H_2_O) and buffer B (2 98% acetonitrile / 0.1% formic acid/ H_2_O). To perform information-dependent acquisition (IDA), the first-order mass spectra were scanned at 250 ms ion accumulation time, and the second-order mass spectra of 30 precursor ions were collected at 50 ms. MS1 spectra were collected in the range of 350–1500 m/z, and MS2 spectra were collected in the range of 100–1500 m/z. The dynamic elimination time of precursor ions was set as 15 s.

### Protein identification and iTRAQ data analysis

In this experiment, the basic process of proteome identification based on mass spectrometry was adopted. After a series of optimized processing, the MS/MS data were scored for protein identification by comparison with proteins in the database. ProteinPilot™ V4.5 search engine (Matrix Science, London, UK; version 2.3.0) matched with AB SCIEX TripleTOF™ 5600 plus was used to identify proteins. The differentially expressed protein was identified and tested for compliance with two criterias: (a) the false discovery rate was less than 1% (the “decoy database searching” was used to estimate the false discovery rate) and (b) protein confidence was more than 95% (“unused ProtScore” ≥ 1.3, −log (1 − % confidence/100) was defined as unused ProtScore). The function and the potential linkage of DEPs were exploited by Gene Ontology (GO) enrichment [17], Kyoto Encyclopedia of Genes and Genomes (KEGG) pathway [18], heatmap and Search Tool for the Retrieval of Interacting Gene (STRING) analyses. We used Blast2GO software to obtain GO annotation and abandon blast results when expected value< 0.001. the GO term was considered when Blast2GO’s score was more than 30. KEGG analysis was used to identify the involved pathways. Pathway enrichment was analysed under Fisher’s exact test.

### Enzyme-linked immunosorbent assay (ELISA)

Human ELISA kits (CUSABIO, Signalway Antibody) were used for further validation which can detect plasma candidate protein levels in another cohort of 100 subjects (50 CoA children and 50 controls). We performed the validation according to manufacturer’s instructions. The validated DEPs met the criterion that (a) proteins expressed differently in CoA and normal children; (b) proteins had underlying pathological or functional significance in CoA; and (c) those proteins have not been previously reported in proteomics study.

### Statistical analysis

Statistical analysis was performed by using GraphPad Prism software (GraphPad Software, San Diego, CA) and SPSS software (SPSS Inc., Chicago, IL, version 17.0). Data are expressed as the mean ± SEM. The values of CoA and the control group were compared using the Mann-Whitney U test. When *p* < 0.05, statistical significance was considered.

## Results

### Demographic data and proteomic analysis

Table 1 shows the demographic data of the CoA children and controls in iTRAQ and ELISA studies, and further clinical information was presented in Supplemental Materials Table 1. After iTRAQ labelling and mass spectrometry analysis, 4975 peptides and 539 proteins were identified. The length of peptides was within the allowable range, with an average of 16.26 amino acids. Among those, 39 proteins were identified as statistically significant, which met both *P* values < 0.05 by Student’s t test and ratios of DEPs > 1.2 or < 0.83. Details of all 39 DEPs have been provided in Supplemental Materials Table 2. Of these, fibulin-1 (FBLN1) and insulin-like growth factor-binding protein complex acid labile subunit (IGFALS or ALS) were considered as possible CoA-associated proteins and were validated by ELISA (Table 2).

**Table 1:** Demographics of study population

	iTRAQ		ELISA	
	CoA	Control	CoA	Control
Number	4	4	50	50
Gender (M%)	2 (50%)	2 (50%)	25 (50%)	25 (50%)
Age (months)	6.97 (4, 8.1)	5.87 (4.5, 7.3)	4.63 ± 2.73	4.08 ± 1.56
Weight (kg)	5.25 (5, 7)	6.33 (5.8, 8)	5.24 ± 0.92	5.18 ± 1.66
Length of CoA (mm)	12.25 (7, 15)	N/A	10.94 ± 5.71	N/A
Diameter of the narrowest (mm)	2.75 (2, 4)	N/A	3.40 ± 1.48	N/A
Type				
Descending aortic arch (N%)		N/A	11 (22%)	N/A
Arch of the aorta (N%)	1 (25%)	N/A	8 (16%)	N/A
Isthmus of the aorta (N%)	3 (75%)	N/A	27 (54%)	N/A
Arch and isthmus of the aorta (N%)		N/A	4 (8%)	N/A

*iTRAQ* isobaric tags for relative and absolute quantitation, *ELISA* enzyme-linked immunosorbent assay, *CoA* Coarctation of the aorta, *M* male, *kg* kilogram, *cm* centimeter, *N* Number, *N/A* not available

**Table 2:** Candidates of differentially expressed proteins in CoA/control Children identified by iTRAQ

Accession	Name	MW (kDa)	Coverage (%)	Unique peptides	Peptides	CoA/control	KEGG Pathway
P2314	FBLN1	77,213.3	61.45	40	40	3.631	TGF-beta signaling pathway
P35858	ALS	66,034.1	8.26	5	5	0.240	ECM-receptor interaction; Hematopoietic cell lineage

*iTRAQ* isobaric tags for relative and absolute quantitation, *MW* molecular weight, *CoA* Coarctation of the aorta, *KEGG* Kyoto Encyclopedia of Genes and Genomes, *FBLN1* Fibulin-1, *ALS* Insulin-like growth factor-binding protein complex acid labile subunit, *TGF* transforming growth factor, *ECM* extracellular matrix

### GO, KEGG, heatmap and STRING analyses

**Fig. 1:**
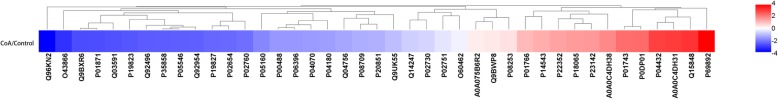
Heatmap of differentially expressed proteins (DEPs). Heatmap represents the ratio levels and functional analysis of all DEPs (*n* = 39) in CoA patients compared with those in control patients. The hierarchical clustering algorithm was based on Euclidean distance. The bottom of the heatmap shows the accession of proteins. The top lines of the heatmap represent similar functions between proteins. The numbers on the right represent the fold change in proteins. The upregulation and downregulation are coloured red and blue, respectively. 24 proteins were down regulated in COA patients, and 15 proteins were up regulated

**Fig. 2:**
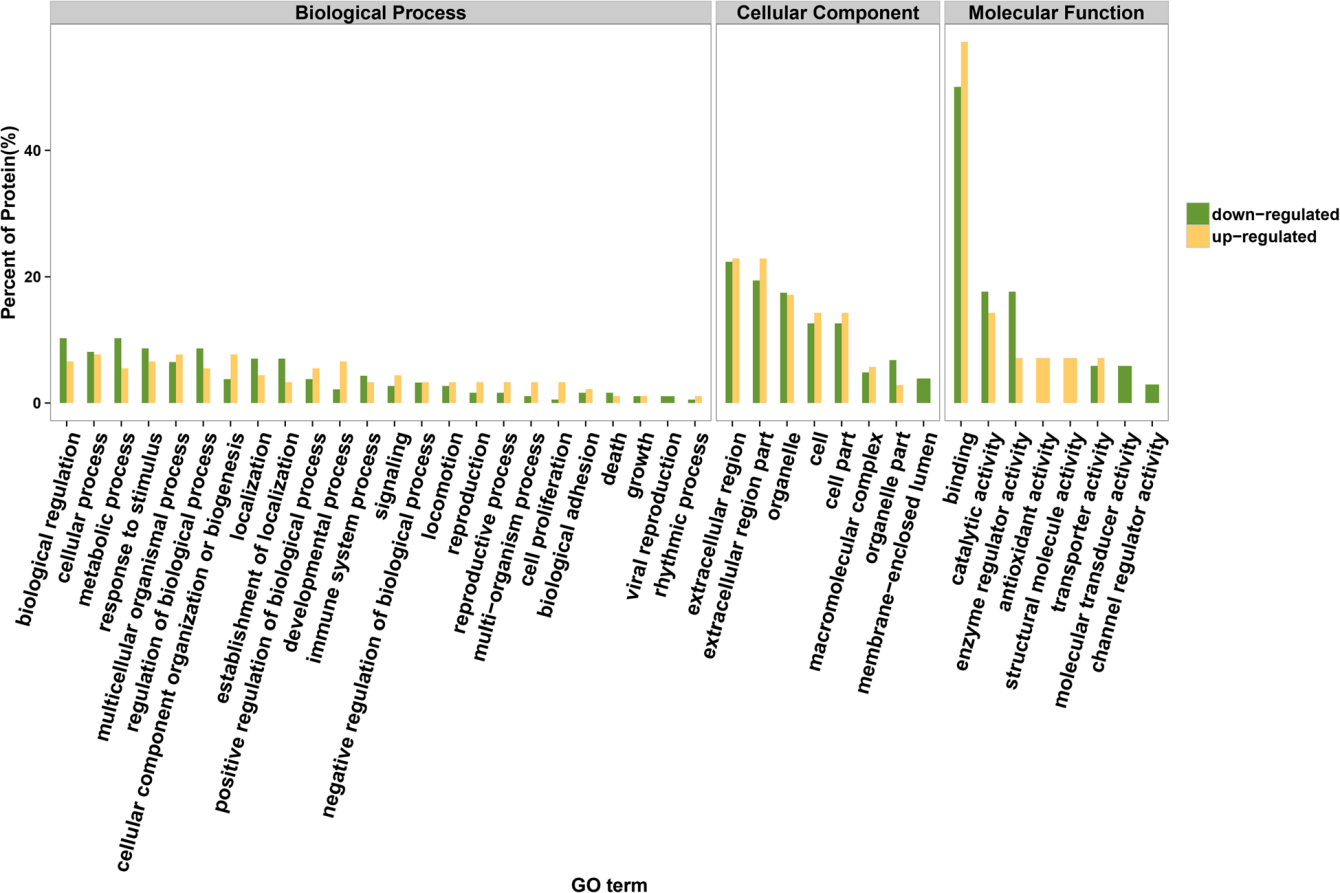
The percentage of proteins in Gene Ontology (GO) terms. GO analysis was an internationally standardized classification system of gene functions, which can describe the properties of genes and gene products. Identified proteins were analysed by GO and represented in three ontologies (biological process, cellular component, and molecular function). The upregulation and downregulation are coloured yellow and green, respectively

**Fig. 3:**
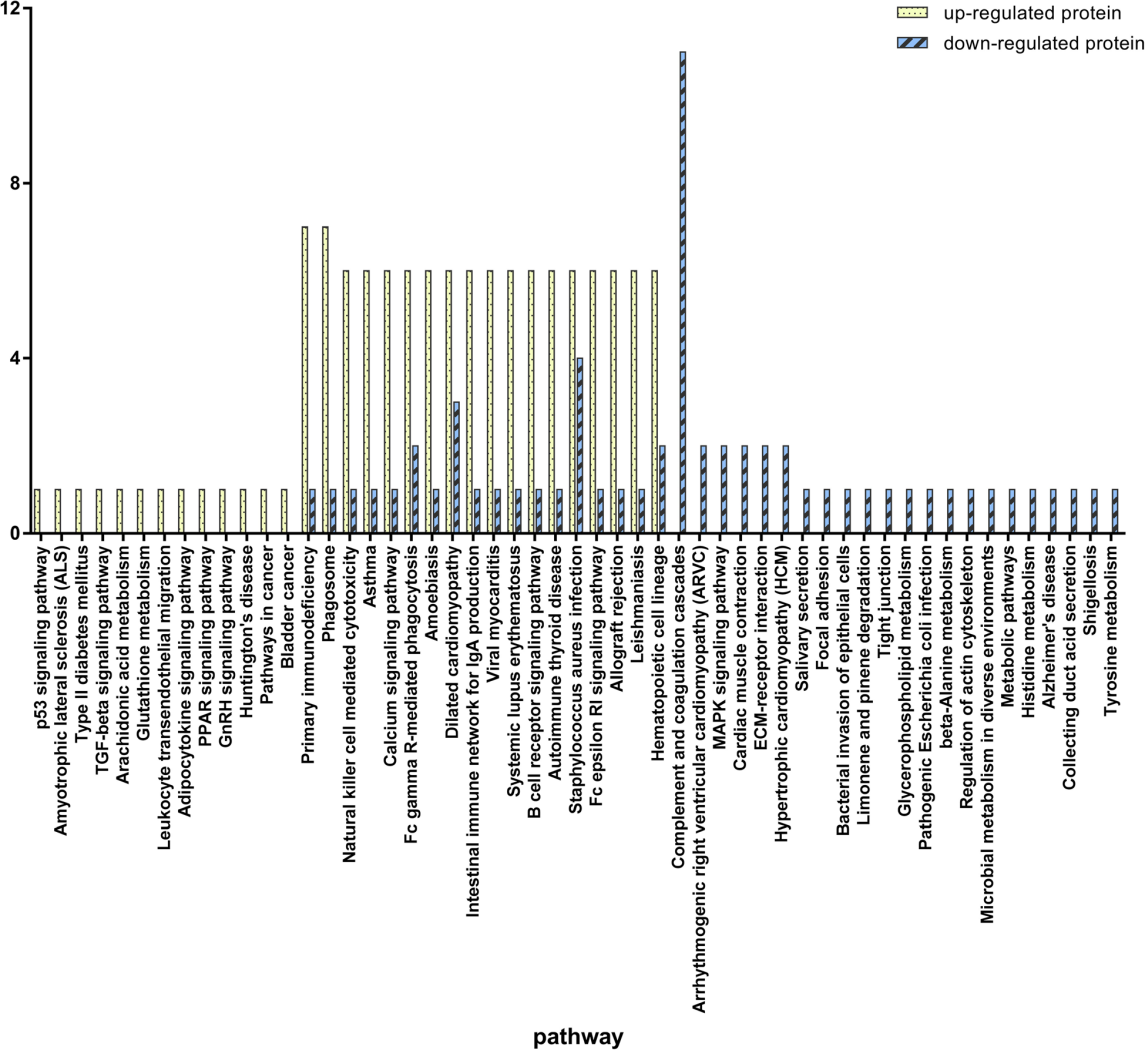
The number of proteins in the Kyoto Encyclopedia of Genes and Genomes (KEGG) pathway. The mainly biochemical metabolic pathways and signal transduction pathways of proteins can be identified by KEGG pathway. All involved KEGG pathways are shown in this figure. The upregulated proteins and downregulated proteins are coloured yellow and blue, respectively

**Fig. 4:**
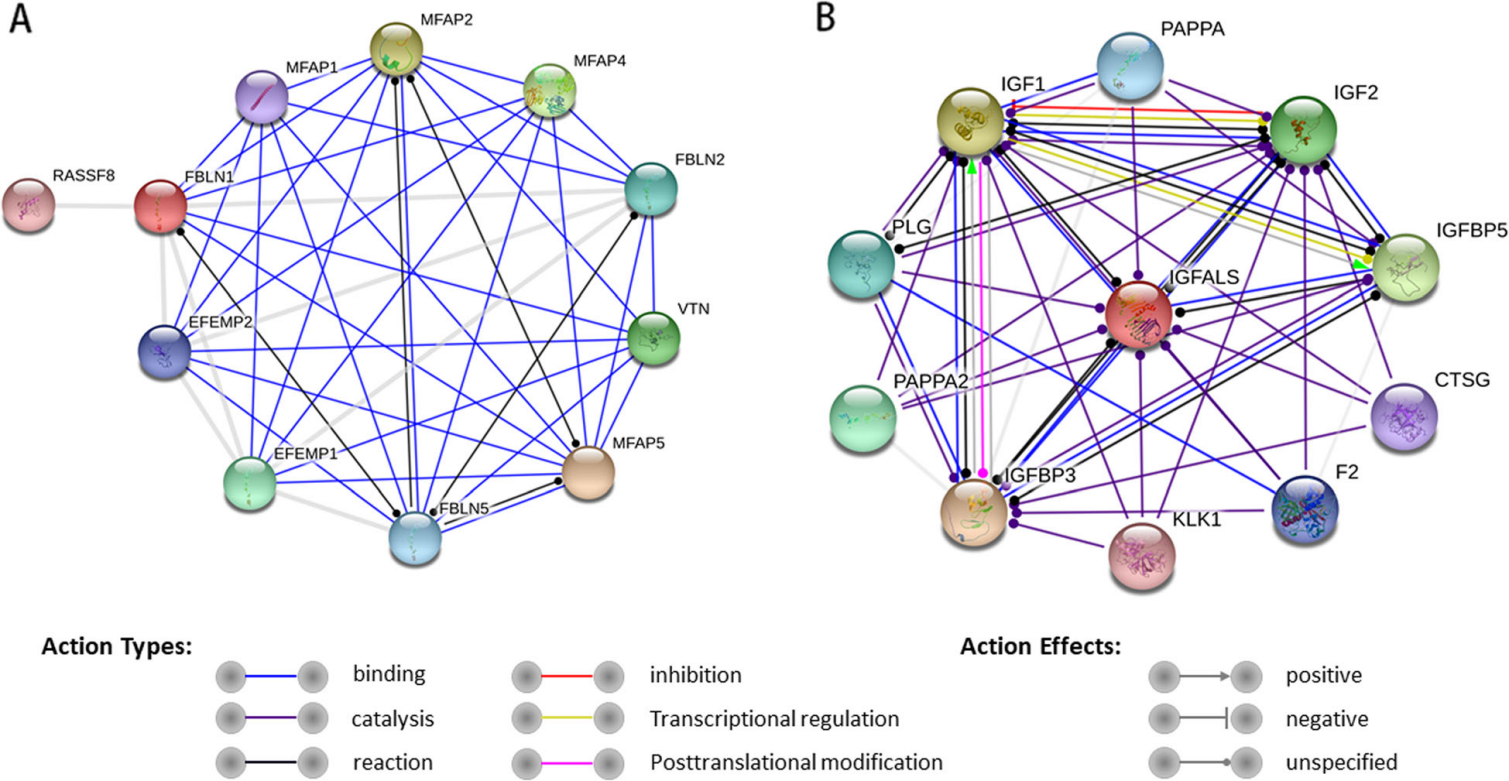
The interaction networks of FBLN1 and ALS predicted by Search Tool for the Retrieval of Interacting Gene (STRING) analysis. The balls represent predicted related proteins, and the lines between the balls represent interactions among the proteins. Different colours represent different interaction types. **a** STRING predicted protein-interaction-network of FBLN1; **b** STRING predicted protein interaction network of ALS

Figure 1 represents the functional analysis and ratio levels of all DEPs in CoA children compared with controls using the hierarchical clustering algorithm which was based on Euclidean distance. Figure 2 shows the percentage of proteins in each GO term in *Homo sapiens*. It was found that: a) the GO term enriching the highest percentage of proteins was binding; b) the GO terms only enriching upregulated proteins were antioxidant activity and structural molecule activity; and c) the GO terms only enriching downregulated proteins were viral reproduction, membrane-enclosed lumen, molecular transducer activity and channel regulator activity. GO analysis indicated that those proteins (Supplemental Materials Table 3) might regulate CoA via influencing the above GO terms. KEGG pathway enrichment exhibited a probable pathway involved in CoA (Fig. 3). STRING analysis was used to predict potential connection between 39 DEPs (Supplemental Materials Figure 1). In addition, we also predicted interaction networks of FBLN1 and ALS (Fig. 4), which could provide more evidence in studying CoA.

### ELISA validation of the candidate proteins

We identified fibulin-1 (FBLN1) and IGFALS (ALS) as candidate proteins and further verified them in the new cohort of CoA children by ELISA (*n* = 50). The reason for choosing the two proteins from 39 DEPs was that they were associated with vascular structure [19], which may correlate to CoA. According to the iTRAQ results, FBLN1 was upregulated in the CoA groups compared to that in the controls, and ALS was downregulated. Further validation by ELISA showed that the level of FBLN1 in the CoA group (8.92 ± 2.36 μg/ml) was significantly higher than that in the control group (6.13 ± 1.94 μg/ml), and the level of ALS in CoA children (348.08 ± 216.74 ng/ml) was significantly lower than the level in normal children (619.46 ± 274.08 ng/ml) (Table 3).

**Table 3:** Validation of candidate proteins by ELISA

	CoA (N = 50)	Control (N = 50)	***P*** value
FBLN1 (μg/ml)	8.92 ± 2.36	6.13 ± 1.94	< 0.001
ALS (ng/ml)	348.08 ± 216.74	619.46 ± 274.08	< 0.001
Hospital stays (days)	30.72 ± 13.00	N/A	
Time of operation (min)	231.30 ± 81.77	N/A	
CPB time (min)	149.64 ± 108.07	N/A	
AO time (min)	56.10 ± 20.57	N/A	
Supporting time of respiratory (h)	69.91 ± 62.60	N/A	
Blood transfusion volume (ml)	593.48 ± 288.41	N/A	

*ELISA* enzyme-linked immunosorbent assay, *CoA* Coarctation of the aorta, *N* number, *FBLN1* Fibulin-1, *ALS* Insulin-like growth factor-binding protein complex acid labile subunit, *CPB* cardiopulmonary bypass, *AO* aortic occlusion, *N/A* not available

## Discussion

Our study was the first to identify the plasma proteins of children with CoA using iTRAQ methods. It was found that 39 proteins were differentially expressed in CoA patients, with 15 upregulated proteins and 24 downregulated proteins. Of these, FBLN1 and ALS, which are related to vascular structure, may be involved in the etiopathogenesis of CoA.

We first chose GO analysis as the break by finding GO terms that only enriched upregulated or downregulated proteins (Supplemental Materials Table 3). It was found that the antioxidant activity and structural molecule activity only enriched upregulated proteins and that viral reproduction, membrane enclosed lumen, molecular transducer activity and channel regulator activity only enriched downregulated proteins. This likely indicated that those GO terms were only activated or inhibited by their enriching proteins in the CoA-related processes. In addition, we further analysed the KEGG pathway of differentially expressed proteins. It was found that most of the DEPs enriched in the four categories of KEGG pathway which were the immunity-related proteins, the coagulation-related proteins, the myocardium-related proteins and the vascular structure-related proteins. Considering the succeeding cardiovascular processes after atrial stenosis, the other categories of KEGG pathway except vascular structure-related proteins may be activated or inhibited after vascular structure alteration; for example, the change in the level of myocardium-related proteins was correlated with elevated LV load [20, 21], and vascular structure alteration can influence immunity [22, 23] and coagulation [24, 25]. Finally, we focused on the vascular structure-related proteins to track the pathogenesis of CoA.

Vascular structure alteration was considered as the initial event of CoA, according to research on the physiopathology of CoA. Substantial evidence has shown that CoA lesions were mainly located in the arterial wall. It was demonstrated by histological examination that a tissue ridge made up of ductal tissue extended from posterior aortic wall into aortic lumen [26]. Increased carotid intima-media thickness was shown in CoA patients [27]. A study focused on the pathophysiological changes in the narrow artery wall and found that elastin fragmenting and collagen deposition increased in the aortic media [6]. In addition, other studies also considered arterial wall injury to exist in CoA patients with both unrepaired and surgically repaired arterial walls [27, 28]. However, the pathogenesis of CoA remains unclear. Three potential mechanisms that may illustrate the pathogenesis of CoA were endothelial cell migration defects, abnormal blood flow and excessive deposition of aortic duct tissue in the aortic isthmus [26]. Thus, our study focused on those proteins that may alter vascular structure to increase the risk of CoA. Finally, FBLN1 and ALS were considered candidates related to CoA after further KEGG pathway analysis (Supplemental Materials Table 3).

FBLN1 expressed in the blood vessel walls and cardiac valves is an important extracellular matrix (ECM) protein during cardiac development and can regulate endothelial-to-mesenchymal transition (EMT) [19, 29–31]. FBLN1 was identified as a vascular stiffness biomarker in recent years. A study of the relationship between FBLN1 and the arterial wall indicated that the level of serum FBLN1 was statistically related to the aortic augmentation index of peripheral arterial disease. The authors also argued that FBLN1 can influence the transforming growth factor-β (TGF-β) pathway to regulate EMT [19], which was verified as the vital role in cardiovascular cells and in many cardiovascular diseases, such as hypertension, cardiac hypertrophy, atherosclerosis and restenosis [32–34]. Thus, we supposed that the level of change in FBLN1 can increase the risk of arterial wall alteration.

ALS is an 85 kDa protein, which was primarily synthesized in the liver and was regulated by growth hormone (GH). ALS can combine with insulin-like growth factor-binding protein (IGFBP) to protect insulin-like growth factor (IGF) from degradation in serum [35, 36]. In contrast, the IGF-IGFBP-ALS complex limits the function of IGF, which must be released from the complex to cross the capillary-endothelial barrier into target tissues [37]. Studies on IGF have shown that it is involved in the migration of human arterial smooth muscle cells (SMCs) [38]; production of nitric oxide in endothelial cells [39, 40]; and some vascular diseases, such as stenotic arteriovenous fistula [41], coronary arteriosclerosis [42], carotid artery intima-media thickening [43] and ischaemic heart disease [44]. In addition, considering that the main effect on IGF release was the proteolysis of IGFBP [36] and that it has been demonstrated in an ALS knockout mouse model that ALS plays a critical role in regulating circulating levels of these proteins [37, 45], we hypothesized that ALS mainly plays a role in biological function by influencing the formation of the IGF-IGFBP-ALS complex. As shown in our results, the ALS level was significantly downregulated in the CoA group compared to the ALS level in the control group. Therefore, we hypothesized that lower levels of ALS could act as IGF-regulators to downregulate the level of IGF-1 and are involved in aortic wall reconstruction in CoA.

However, our study also exhibited some deficiencies. Based on proteomic technology, our results simply presented the plasma level of protein changes, and the mRNA expression needs further validation. In addition, the two proteins were considered as CoA-related candidates by analysing the results of iTRAQ and ELISA and by making conclusions based on the current studies about other vascular diseases; the vital matter, such as TGF-β and the IGF-IGFBP-ALS complex, should be detected to critically verify their relation to CoA. Further, deeper research on the function of these proteins should be performed.

## Conclusion

In conclusion, our study was the first to identify differentially expressed proteins by using the iTRAQ method in CoA children, which may explain the underlying mechanisms of CoA. Our results showed that vascular structure alteration was the key event in the initiation and progression of CoA. FBLN1 and ALS are critical proteins that may act as biomarkers for diagnosing CoA and estimating the conditions of prognosis of CoA in the clinic. These findings may provide a basis for advancing the understanding of the aetiology and pathogenesis of CoA.

## Supplementary information

**Additional file 1.**

**Additional file 2: Table S1**. Clinical information of CoA patients.

**Additional file 3: Table S2.** Differentially expressed proteins in CoA/control Children identified by iTRAQ.

**Additional file 4: Table S3**. KEGG Pathway of proteins.

## Data Availability

Please contact the author for data requests.
