# Supplementary figures and images for: Identified plasma proteins related to vascular structure are associated with coarctation of the aorta in children
Source: Ital J Pediatr. 2020 May 19;46:63. doi: 10.1186/s13052-020-00830-7 (PMC7236479; doi:10.1186/s13052-020-00830-7)

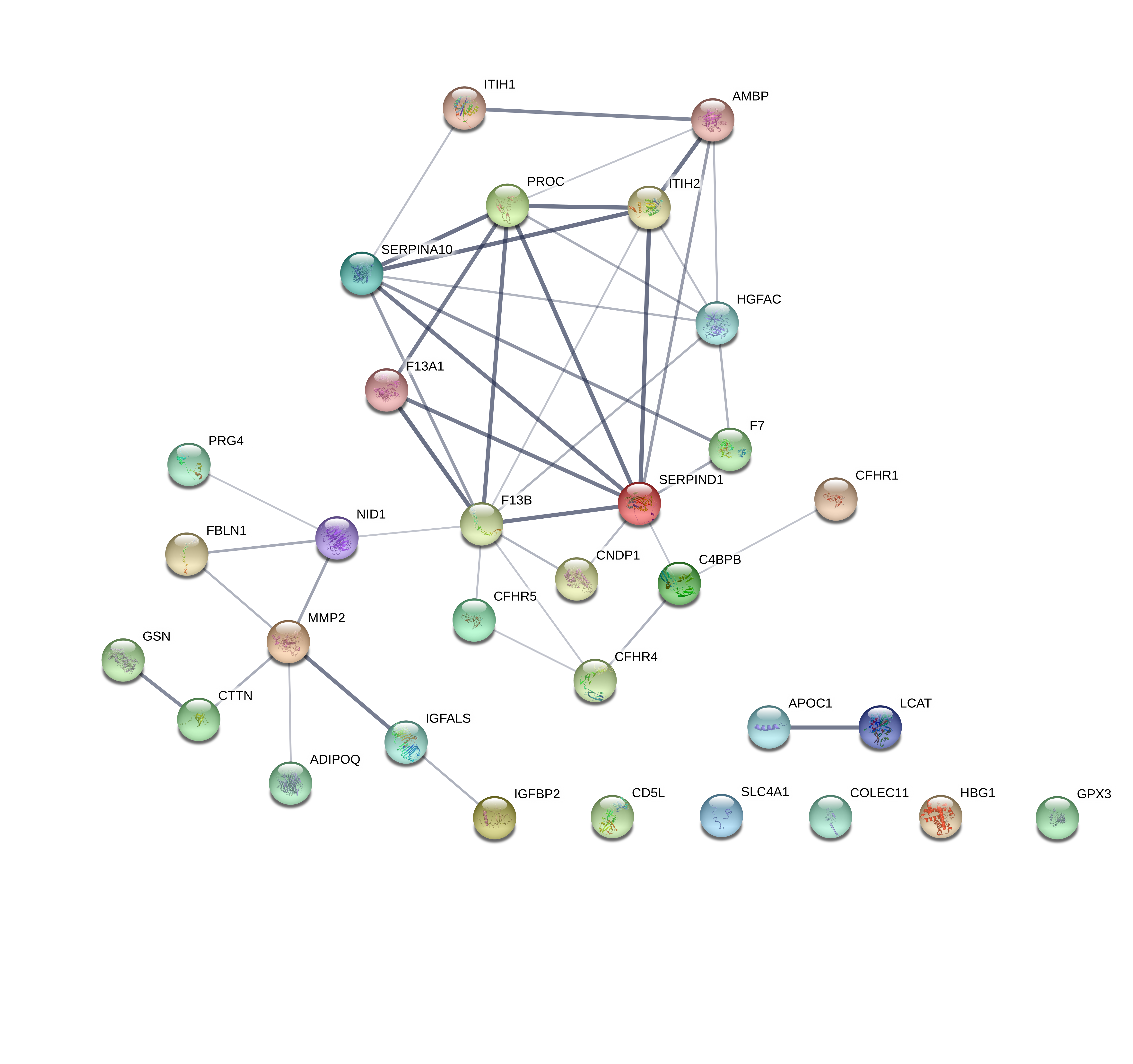

Supplement: Supplementary file 1 — Additional file 1. [file 13052_2020_830_MOESM1_ESM.tif]
