# Supplementary material for: Identified plasma proteins related to vascular structure are associated with coarctation of the aorta in children
Source: Ital J Pediatr. 2020 May 19;46:63. doi: 10.1186/s13052-020-00830-7 (PMC7236479; doi:10.1186/s13052-020-00830-7)
Supplement: Supplementary file 2 — Additional file 2: Table S1. Clinical information of CoA patients. [file 13052_2020_830_MOESM2_ESM.docx]

Supplemental material table 1: Clinical information of CoA patients.

| **Patient number** | **Other congenital heart disease** | **Other non-congenital heart disease** |
| --- | --- | --- |
| 1 | Aortic arch hypoplasia; VSD; PDA; PH | None |
| 2 | Aortic arch hypoplasia; VSD; PDA; PFO; PH | None |
| 3 | Aortic arch hypoplasia; VSD; Mitral valve anterior lobe; MI; TVP; TI; PFO; PH | None |
| 4 | Aortic arch hypoplasia; VSD; Bicuspid aortic valve; PH | Severe pneumonia; Respiratory insufficiency; Cardiac insufficiency |
| 5 | VSD; PDA; PFO; PH | Cardiac insufficiency; Stenosis of left main bronchus; Bronchopneumonia |
| 6 | VSD; ASD | Bronchopneumonia; Left lower atelectasis |
| 7 | Aortic arch hypoplasia; VSD; ASD; PH | Severe pneumonia; Cardiac insufficiency; Respiratory insufficiency |
| 8 | Aortic arch hypoplasia; VSD; PFO; PH | Pulmonary infection; Cardiac insufficiency; Left oblique inguinal hernia |
| 9 | VSD; PDA; PFO; PH | Cardiac insufficiency; Bronchopneumonia; Mild anemia; Hyperbilirubinemia; Chylous ascites |
| 10 | ASD; MR; TR; PH | Cardiac insufficiency; Pericardial effusion; Bronchopneumonia |
| 11 | Aortic arch hypoplasia; Collateral circulation of descending aorta | None |
| 12 | Aortic arch hypoplasia; MI | Cardiac insufficiency |
| 13 | Aortic arch hypoplasia; VSD; ASD; MI; TI; PH | Cardiac insufficiency; Neonatal pneumonia |
| 14 | VSD; PFO; PH | Neonatal jaundice; Cardiac insufficiency; Respiratory insufficiency; Bronchopneumonia |
| 15 | Aortic arch hypoplasia; VSD; ASD; PDA; PH | None |
| 16 | Aortic arch hypoplasia; VSD; MI; TI; PFO; PH | Bronchopneumonia; Cardiac insufficiency; Respiratory insufficiency; Severe pneumonia; Anemia |
| 17 | Aortic arch hypoplasia; Bicuspid aortic valve with AS | None |
| 18 | Aortic arch hypoplasia; VSD; ASD; PDA; PH | Down's syndrome |
| 19 | VSD; ASD; PH | Bronchopneumonia; Cardiac insufficiency; Respiratory insufficiency |
| 20 | Aortic arch hypoplasia; PDA; PH; Left vertebral artery of aortic arch origin | Psychomotor retardation |
| 21 | PDA | None |
| 22 | Aortic arch hypoplasia; VSD; Abnormal origin of right coronary artery; PFO; PH | Bronchopneumonia |
| 23 | VSD; ASD; PH; MI; TI; PAPVC | None |
| 24 | Aortic arch hypoplasia; ASD; MVP; MI; PH | Cardiac insufficiency; Respiratory insufficiency; Congenital hypoplasia of laryngeal cartilage |
| 25 | Aortic arch hypoplasia; PDA; ASD; PH | None |
| 26 | Aortic arch hypoplasia; VSD; ASD; PH | Neonatal pneumonia; Cardiac insufficiency; Respiratory insufficiency; Subdural effusion; Scalp hematoma |
| 27 | VSD; ASD; PH | Premature pneumonia; Bronchostenosis; Cardiac insufficiency; Atelectasis; Respiratory insufficiency |
| 28 | Aortic arch hypoplasia; PDA; PH | None |
| 29 | Aortic arch hypoplasia; PDA | None |
| 30 | Aortic arch hypoplasia; VSD; MI; TI; PFO; PH | None |
| 31 | VSD | Incomplete right bundle branch block |
| 32 | VSD; ASD; Right vagal subclavian artery; PH | Growth retardation |
| 33 | PDA; MI; PFO; PH | Severe pneumonia; Cardiac insufficiency; Respiratory insufficiency |
| 34 | Aortic arch hypoplasia; VSD; ASD; PDA; PH | Severe pneumonia; Cardiac insufficiency; Respiratory insufficiency; Incomplete right bundle branch block |
| 35 | ASD; PH | Cardiac insufficiency |
| 36 | Aortic arch hypoplasia; VSD; PFO; PDA; PH | Neonatal pneumonia; Cardiac insufficiency; Respiratory insufficiency; Intracranial hemorrhage |
| 37 | VSD; PFO; PH | Left main bronchial stenosis |
| 38 | Aortic arch hypoplasia; VSD; ASD; PDA; PH | Bronchopneumonia; Cardiac insufficiency; Respiratory insufficiency; Left main bronchial stenosis |
| 39 | VSD; ASD; PDA; MI; TI; PH | Bronchopneumonia; Cardiac insufficiency; Respiratory insufficiency |
| 40 | VSD; ASD; PH | Neonatal pneumonia; Cardiac insufficiency; Respiratory insufficiency; Intracranial hemorrhage; congenital cleft palate; Seroperitoneum; Renal laceration |
| 41 | PLSVC | Premature ventricular contraction; Psychomotor retardation; Cardiac insufficiency; Respiratory insufficiency; Thrush; Inherited metabolic disorders |
| 42 | PDA; Bicuspid aortic valve; PH | Right tracheal bronchus |
| 43 | Aortic arch hypoplasia; RVOTO; PLSVC; Left vagal vertebral artery | Left anterior fascicular block |
| 44 | PDA | None |
| 45 | VSD; PH | None |
| 46 | VSD; ASD; PDA; Malformation of coronary artery; PH | Gastric volvulus |
| 47 | ASD | Deafness |
| 48 | LVOTO; VSD; PDA | None |
| 49 | Bicuspid aortic valve with AS | None |
| 50 | MI | Cardiac insufficiency; Respiratory insufficiency; Hemangioma of the left upper eyelid and oral cavity |
| 51 | Aortic arch hypoplasia | None |
| 52 | Aortic arch hypoplasia; ASD; Mitral valve deformity with regurgitation; PH | Cardiac insufficiency |
| 53 | Aortic arch hypoplasia; VSD; ASD; PDA; Right-sided aortic arch | Neonatal pneumonia; Liver damage; Cardiac insufficiency; Respiratory insufficiency |
| 54 | VSD; PDA; PH | Bronchostenosis |

Note. VSD: ventricular septal defect; PDA: patent ductus arteriosus; PH: pulmonary hypertension; PFO: patent foramen ovale; MI: mitral insufficiency; MR: mitral regurgitation; TVP: tricuspid valve prolapse; TI: tricuspid insufficiency; TR: tricuspid regurgitation; ASD: atrial septal defect; AS: aortic stenosis; PAPVC: partial anomalous pulmonary venous connection; MVP: mitral valve prolapse; PLSVC: perpetuate the left superior vena cava; RVOTO: right ventricular outflow tract obstacle; LVOTO: left ventricular outflow tract obstacle;
