# Supplementary material for: Identified plasma proteins related to vascular structure are associated with coarctation of the aorta in children
Source: Ital J Pediatr. 2020 May 19;46:63. doi: 10.1186/s13052-020-00830-7 (PMC7236479; doi:10.1186/s13052-020-00830-7)
Supplement: Supplementary file 3 — Additional file 3: Table S2. Differentially expressed proteins in CoA/control Children identified by iTRAQ. [file 13052_2020_830_MOESM3_ESM.docx]

Supplemental material table 2: Differentially expressed proteins in CoA/control Children identified by iTRAQ

| **Accession** | **Name** | **%Coverage (95)** | **Peptides** | **Unique peptides** | **MW (kDa)** | **Protein Length** | **CoA/control** |
| --- | --- | --- | --- | --- | --- | --- | --- |
| P69892 | Hemoglobin subunit gamma-2 | 20.40999979 | 3 | 2 | 16126.3 | 147 | 17.06453186 |
| Q15848 | Adiponectin | 38.93250078 | 8 | 8 | 26413.6 | 244 | 10.20505505 |
| A0A0C4DH31 | Immunoglobulin heavy variable 1-18 | 61.53750047 | 7 | 3 | 12820.4 | 117 | 9.359068086 |
| P04432 | Immunoglobulin kappa variable 1D-39 | 52.9949978 | 7 | 3 | 12737.3 | 117 | 8.895441186 |
| P0DP01 | Immunoglobulin heavy variable 1-8 | 72.65333334 | 9 | 5 | 12991.6 | 117 | 5.717116513 |
| P01743 | Immunoglobulin heavy variable 1-46 | 66.45499915 | 8 | 4 | 12932.6 | 117 | 5.621750099 |
| A0A0C4DH38 | Immunoglobulin heavy variable 5-51 | 54.91000042 | 5 | 5 | 12674.4 | 117 | 4.238424377 |
| P2314 | Fibulin-1 | 60.74000001 | 37 | 37 | 77213.3 | 703 | 3.631271894 |
| P18065 | Insulin-like growth factor-binding protein 2 | 16.22999981 | 3 | 3 | 34813.8 | 325 | 3.521401455 |
| P22352 | Glutathione peroxidase 3 | 44.2475006 | 12 | 12 | 25552.2 | 226 | 3.27292507 |
| P14543 | Nidogen-1 | 17.5999999 | 14 | 14 | 136375.9 | 1247 | 2.912363913 |
| P01766 | Immunoglobulin heavy variable 3-13 | 71.33750021 | 8 | 4 | 12506.1 | 116 | 2.575616441 |
| P08253 | 72 kDa type IV collagenase | 25.19000061 | 12 | 12 | 73881.7 | 660 | 1.71116682 |
| Q9BWP8 | Collectin-11 | 31.08999953 | 8 | 8 | 28665.2 | 271 | 1.596252704 |
| A0A075B6R2 | Immunoglobulin heavy variable 4-4 | 35.32666564 | 4 | 1 | 12847.6 | 117 | 1.513604088 |
| P02730 | Band 3 anion transport protein | 1.893500053 | 1 | 1 | 101791.3 | 911 | 0.810951619 |
| Q14247 | Src substrate cortactin | 5.454999954 | 2 | 2 | 61585.9 | 550 | 0.762151785 |
| Q9UK55 | Protein Z-dependent protease inhibitor | 27.8700009 | 11 | 11 | 50706.6 | 444 | 0.640204412 |
| P20851 | C4b-binding protein beta chain | 54.95999754 | 14 | 14 | 28357.2 | 252 | 0.563025848 |
| P08709 | Coagulation factor VII | 31.43999949 | 9 | 9 | 51593.5 | 466 | 0.547668073 |
| Q04756 | Hepatocyte growth factor activator | 20.19249983 | 9 | 9 | 70681.1 | 655 | 0.50523401 |
| P04180 | Phosphatidylcholine-sterol acyltransferase | 21.42250017 | 7 | 7 | 49577.5 | 440 | 0.464937263 |
| P04070 | Vitamin K-dependent protein C | 41.53999984 | 15 | 14 | 52070.8 | 461 | 0.44522443 |
| P06396 | Gelsolin | 25.63749999 | 13 | 13 | 85696.9 | 782 | 0.434979305 |
| P00488 | Coagulation factor XIII A chain | 36.85000018 | 24 | 24 | 83266.8 | 732 | 0.43121218 |
| P05160 | Coagulation factor XIII B chain | 44.0624997 | 23 | 23 | 75510.1 | 661 | 0.36337132 |
| P02760 | Protein AMBP | 28.19750011 | 9 | 9 | 38999.2 | 352 | 0.285154383 |
| P02654 | Apolipoprotein C-I | 41.26499966 | 11 | 11 | 9331.8 | 83 | 0.271100252 |
| P19827 | Inter-alpha-trypsin inhibitor heavy chain H1 | 18.98999997 | 12 | 12 | 101388.5 | 911 | 0.266306663 |
| Q92954 | Proteoglycan 4 | 7.994499896 | 8 | 8 | 151075.8 | 1404 | 0.243203834 |
| P05546 | Heparin cofactor 2 | 13.37749995 | 7 | 7 | 57070.2 | 499 | 0.240968878 |
| P35858 | Insulin-like growth factor-binding protein complex acid labile subunit | 14.50350005 | 5 | 5 | 66034.1 | 605 | 0.239958978 |
| Q92496 | Complement factor H-related protein 4 | 17.25999974 | 7 | 7 | 65350.2 | 578 | 0.235750256 |
| P19823 | Inter-alpha-trypsin inhibitor heavy chain H2 | 25.34499988 | 18 | 18 | 106462.7 | 946 | 0.222459843 |
| Q03591 | Complement factor H-related protein 1 | 63.10500056 | 21 | 7 | 37650.3 | 330 | 0.210725054 |
| P01871 | Immunoglobulin heavy constant mu | 69.53750104 | 51 | 16 | 49439.4 | 453 | 0.202435487 |
| Q9BXR6 | Complement factor H-related protein 5 | 36.81749925 | 17 | 15 | 64419 | 569 | 0.200759504 |
| O43866 | CD5 antigen-like | 49.92750064 | 15 | 15 | 38087.6 | 347 | 0.151138091 |
| Q96KN2 | Beta-Ala-His dipeptidase | 30.81749976 | 11 | 11 | 56705.6 | 507 | 0.091513166 |

Note. iTRAQ: isobaric tags for relative and absolute quantitation; CoA: Coarctation of the aorta; MW: molecular weight.
