# Supplementary material for: Identified plasma proteins related to vascular structure are associated with coarctation of the aorta in children
Source: Ital J Pediatr. 2020 May 19;46:63. doi: 10.1186/s13052-020-00830-7 (PMC7236479; doi:10.1186/s13052-020-00830-7)
Supplement: Supplementary file 4 — Additional file 4: Table S3. KEGG Pathway of proteins. [file 13052_2020_830_MOESM4_ESM.docx]

Supplemental material table 3: KEGG Pathway of proteins.

| **GO Function** | **Accession** | **Name** | **Abbreviation** | **CoA/control** | **Differential expression** | **KEGG Pathway** |
| --- | --- | --- | --- | --- | --- | --- |
| antioxidant activity | P22352 | Glutathione peroxidase 3 | GPX3 | 3.27292507 | up | Glutathione metabolism;  Amyotrophic lateral sclerosis;  Arachidonic acid metabolism;  Huntington's disease |
| structural molecule activity | P23142 | Fibulin-1 | FBLN1 | 3.631271894 | up | TGF-beta signaling pathway |
| viral reproduction | P06396 | Gelsolin | GELS | 0.434979305 | down | Regulation of actin cytoskeleton;  Fc gamma R-mediated phagocytosis |
| viral reproduction & channel regulator activity | P02760 | Protein AMBP | AMBP | 0.285154383 | down | Complement and coagulation cascades |
| membrane-enclosed lumen | P04070 | Vitamin K-dependent protein C | PROC | 0.44522443 | down | Complement and coagulation cascades |
| membrane-enclosed lumen | P08709 | Coagulation factor VII | FA7 | 0.547668073 | down | Complement and coagulation cascades |
| membrane-enclosed lumen | P00488 | Coagulation factor XIII A | F13A | 0.43121218 | down | Complement and coagulation cascades |
| membrane-enclosed lumen | P35858 | Insulin-like growth factor-binding protein complex acid labile subunit | ALS | 0.239958978 | down | ECM-receptor interaction;  Hematopoietic cell lineage |
| transporter activity | Q92496 | Complement factor H-related protein 4 | FHR4 | 0.235750256 | down | Complement and coagulation cascades;  Staphylococcus aureus infection |
| transporter activity | P02730 | Band 3 anion transport protein | B3AT | 0.810951619 | down | Collecting duct acid secretion |

Note. GO: Gene Ontology; KEGG: Kyoto Encyclopedia of Genes and Genomes.
